# Cellars

**Published:** 1868-08

**Authors:** 


### CELLARS.

THERE ought to be no cellar under any building designed for the residence of families; but in cities where ground is very valuable it is considered a necessity. The only access to the cellar of a New-York dwelling is through the lower hall or passage; hence, whenever the door is opened, all the fumes, gases, odors and damps which arise from it, ascend through the building and impregnate every room with the foul air generated by decaying wood, vegetables, bones, skins, scraps of meat, etc., of which the cellar is too commonly made a common receptacle.

In the spring or early summer, every movable thing should be taken out, the walls and floor should be most thoroughly swept, and then the walls and ceiling should be most profusely whitewashed. No cellar should be without a well-plastered ceiling, not only to exclude the dampness and bad air, but to protect the lower room from cold and changeable weather.

There can be no doubt that an ill-conditioned cellar is the unsuspected cause of death among many a happy household. A gentleman recently built for himself a splendid mansion in this city. He had not been in it long before several members of a hitherto healthy family became unwell. On minute inquiry he found that the house had been erected on a filled-in swampy lot. He at once removed elsewhere, and the usual health returned to his household.

During one of the cholera years in Boston, the whole city was divided into small districts, and trusty citizens were appointed to visit each, and to leave no part of any suspicious premises unexamined, with power to compel a thorough and immediate cleansing. Their care was happily rewarded: only in one neighborhood was there any marked disease, when upon a more rigid exploration of a particular building, it was found that one compartment of a dark cellar was almost filled with a disgusting compound of all the offal of a kitchen for a long period. With such facts, those who possess any intelligence, with even a moderate affection for their wives and children, will give a prompt and wise attention to the subject.

